# Suppression of homology-dependent DNA double-strand break repair induces PARP inhibitor sensitivity in *VHL*-deficient human renal cell carcinoma

**DOI:** 10.18632/oncotarget.23470

**Published:** 2017-12-19

**Authors:** Susan E. Scanlon, Denise C. Hegan, Parker L. Sulkowski, Peter M. Glazer

**Affiliations:** ^1^ Department of Therapeutic Radiology, Yale University School of Medicine, New Haven, CT, USA; ^2^ Department of Experimental Pathology, Yale University School of Medicine, New Haven, CT, USA; ^3^ Department of Genetics, Yale University School of Medicine, New Haven, CT, USA

**Keywords:** DNA repair, homologous recombination, von Hippel-Lindau (VHL), hypoxia, PARP inhibitor

## Abstract

The von Hippel-Lindau (*VHL*) tumor suppressor gene is inactivated in the vast majority of human clear cell renal carcinomas. The pathogenesis of *VHL* loss is currently best understood to occur through stabilization of the hypoxia-inducible factors, activation of hypoxia-induced signaling pathways, and transcriptional reprogramming towards a pro-angiogenic and pro-growth state. However, hypoxia also drives other pro-tumorigenic processes, including the development of genomic instability via down-regulation of DNA repair gene expression. Here, we find that DNA repair genes involved in double-strand break repair by homologous recombination (HR) and in mismatch repair, which are down-regulated by hypoxic stress, are decreased in *VHL*-deficient renal cancer cells relative to wild type *VHL*-complemented cells. Functionally, this gene repression is associated with impaired DNA double-strand break repair in *VHL*-deficient cells, as determined by the persistence of ionizing radiation-induced DNA double-strand breaks and reduced repair activity in a homology-dependent plasmid reactivation assay. Furthermore, *VHL* deficiency conferred increased sensitivity to PARP inhibitors, analogous to the synthetic lethality observed between hypoxia and these agents. Finally, we discovered a correlation between *VHL* inactivation and reduced HR gene expression in a large panel of human renal carcinoma samples. Together, our data elucidate a novel connection between *VHL*-deficient renal carcinoma and hypoxia-induced down-regulation of DNA repair, and identify potential opportunities for targeting DNA repair defects in human renal cell carcinoma.

## INTRODUCTION

The von Hippel-Lindau (*VHL*) gene is mutated, deleted, or silenced in 60–80% of human clear cell renal cell carcinomas (ccRCC), the most common type of kidney cancer [1]. Reintroduction of wild-type *VHL* into *VHL*-deficient renal carcinoma cells suppresses tumor formation, establishing its role as a tumor suppressor [2]. The best-characterized tumor suppressor function of the *VHL* protein (pVHL) is as the substrate-recognition subunit of an E3 ubiquitin ligase that targets hypoxia-inducible factor (HIF) α-subunits for degradation under normoxic conditions [3, 4]. HIF stability is regulated by oxygen tension due to the activity of oxygen-dependent prolyl hydroxylases (PHDs) (reviewed in [5–7]). In the presence of adequate oxygen, the PHDs hydroxylate HIF α-subunits at proline residues, which allows their binding by pVHL, polyubiquitination by the pVHL/Elongin B/Elongin C/Cul 2/Rbx-1 E3 ligase complex, and degradation by the proteasome. Under low oxygen conditions, the PHDs are inhibited, preventing recognition of the HIF α-subunits by pVHL. Analogously, inactivation of pVHL results in constitutive HIF α-subunit stabilization. The stabilized HIF α-subunits dimerize with a constitutively-expressed HIF β-subunit, translocate to the nucleus, and induce changes in gene transcription by recruiting the p300/CBP transcriptional activators to consensus genetic sequences termed hypoxia response elements. The transcriptional reprogramming induced by the two primary HIF isoforms, HIF-1 and HIF-2, promotes angiogenesis, cell proliferation, and changes in energy metabolism, which aid in the adaptation to hypoxia, but can also promote tumorigenesis when dysregulated. HIF stabilization and activation of target genes is critical in the pathogenesis of ccRCC as HIF-2 has been shown to be both necessary and sufficient for tumor growth in *VHL*-deficient RCC cell lines [8–11].

Independent of *VHL* deficiency, hypoxia is an important physiological component of solid tumors and contributes to tumor progression and metastasis (reviewed in [12–15]). Hypoxia, which is found in most solid tumors, correlates with aggressive tumor features and also serves as an independent prognostic indicator of poor patient outcome in several cancer types. One of the mechanisms by which hypoxia promotes tumorigenesis is the induction of genomic instability, which is recognized as a key enabling characteristic of cancer [16]. Hypoxia has been shown, both *in vitro* and *in vivo*, to lead to a wide range of genetic alterations, including elevated mutation frequency, DNA over-replication, fragile site induction, and microsatellite instability [17–21].

Although hypoxia induces genomic instability, it does not directly generate DNA damage. Instead, hypoxic stress facilitates the down-regulation of cellular DNA repair pathways through transcriptional, translational, and epigenetic mechanisms (reviewed in [22–24]). DNA double-strand break repair by homologous recombination (HR) has been shown to be repressed under hypoxia via the coordinated down-regulation of BRCA1, RAD51, and FANCD2 by the E2F4/p130 transcription repressor complex [25–28]. Similarly, mismatch repair (MMR) is decreased by hypoxic stress via the down-regulation of MLH1 and MSH2 by the Myc/Max transcription factor network [29, 30]. HR and MMR have also been shown to be down-regulated via reduced translation efficiency or microRNA inhibition of translation of HR and MMR proteins [31–33]. Moreover, hypoxia can lead to stable silencing of the *BRCA1* and *MLH1* gene promoters [34, 35]. Nucleotide excision repair and base excision repair are also reduced under hypoxia via decreased transcription or translation of key protein factors in these pathways [36, 37]. In contrast, the error-prone DNA double-strand break repair pathway of non-homologous end joining (NHEJ) does not appear to be a consistent target of hypoxic stress, with some studies indicating increased, decreased, or no change in expression of NHEJ genes and in NHEJ activity [26, 38–40]. Hypoxia within solid tumors is currently being investigated as a potential therapeutic target, as cells with reduced DNA repair capacity are susceptible to DNA damaging agents or inhibition of complementary DNA repair pathways. Specifically, hypoxic cells have been shown to exhibit synthetic lethality with poly-ADP-ribose polymerase (PARP) inhibitors [41, 42].

Given the similar downstream effects of *VHL* mutations and physiologic hypoxia, we hypothesized that *VHL*-deficient RCC may have reduced DNA repair capacity that could be exploited for therapeutic gain. Interestingly, several possible connections between pVHL and DNA repair have been reported. First, pVHL has been shown to positively regulate p53 by inhibiting Mdm2-mediated p53 ubiquitination [43, 44]. Loss of pVHL thus leads to reduced p53 activation, impaired cell cycle checkpoint activation, and in some cases, reduced apoptosis in response to DNA damage. pVHL has also been shown to localize to the mitotic spindle and to prevent spindle misorientation and aneuploidy [45]. Recently, a direct role for pVHL in DNA double-strand break repair by HR has been shown to be dependent on SOCS1-mediated K63-ubiquitination of pVHL and its redistribution to nuclear foci [46]. Finally, *VHL*-deficient cells have been shown to have reduced nucleotide excision repair (NER) capacity [47]. Thus, *VHL* clearly plays a role in maintaining genomic stability, but whether the mechanisms induced by hypoxia leading to coordinated down-regulation of DNA repair pathways occur in *VHL*-deficient cells has not yet been investigated.

In this study, we have investigated the possibility that *VHL* mutations, through induction of hypoxia-like signaling pathways, may lead to down-regulation of DNA repair pathways and sensitivity to DNA damage. We have found that *VHL*-deficient human renal carcinoma cells have reduced protein and mRNA expression of key HR and MMR genes down-regulated by hypoxia, including *BRCA1*, *RAD51*, *FANCD2*, and *MLH1*. Using siRNA depletion, we have demonstrated that this reduced gene expression is directly linked to loss of pVHL. We have further established that this decrease in HR gene expression is associated with reduced repair of DNA double-strand breaks by HR and consequent sensitivity to PARP inhibitors in *VHL*-deficient renal carcinoma cells. Finally, by analyzing mRNA expression in human renal carcinoma samples in The Cancer Genome Atlas (TCGA), we have identified a correlation between *VHL* deficiency in RCC and reduced expression of HR and MMR genes, supporting the significance of our findings in human RCC.

## RESULTS

### *VHL*-deficient renal cells have reduced expression of homologous recombination and mismatch repair genes

**Figure 1:**
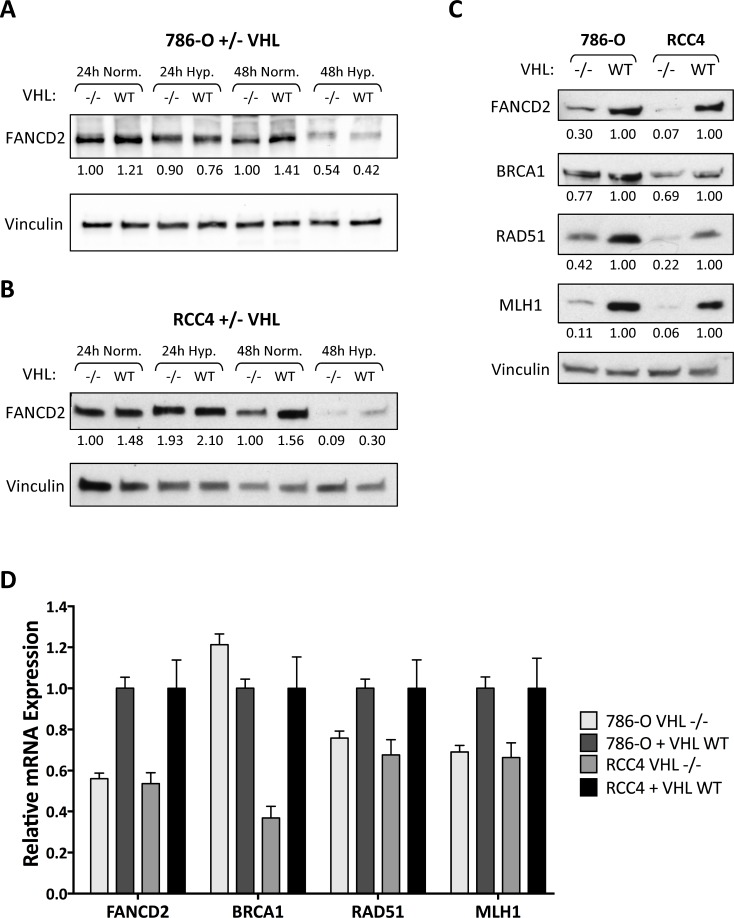
HR and MMR protein and mRNA expression is reduced in *VHL*-deficient renal carcinoma cells (**A**, **B**) Western blotting was performed to measure FANCD2 protein expression in 786-O^VHL–/–^ and 786-O+VHL^WT^ cells (A) or RCC4^VHL–/–^ and RCC4+VHL^WT^ cells (B) exposed to normoxia or hypoxia (<0.01% O_2_) for 24 or 48 h. Vinculin is presented as loading control and numbers below the blots indicate FANCD2 band density relative to vinculin and normalized to 786-O^VHL–/–^ or RCC4^VHL–/–^ normoxia samples. (**C**) Western blotting for additional DNA repair proteins was performed in untreated 786-O and RCC4 matched pair cells. Vinculin is presented as loading control and numbers below the blots indicate band density relative to vinculin and normalized to VHL^WT^ samples. (**D**) qRT-PCR was performed to measure *FANCD2*, *BRCA1*, *RAD51*, and *MLH1* mRNA levels in untreated 786-O and RCC4 matched pair cells. Expression levels were normalized to 18S rRNA expression and presented as relative to VHL^WT^ samples. *Columns*, mean of 3 replicates; *bars*, SEM.

We began by investigating the regulation of DNA repair pathways in two *VHL*-deficient human ccRCC cell lines, 786-O^VHL–/–^ and RCC4^VHL–/–^, and their wild-type *VHL*-complemented matched pairs, 786-O+VHL^WT^ and RCC4+VHL^WT^. We confirmed that these cells overexpress the HIF α-subunits (HIF-2α in 786-O^VHL–/–^ cells and both HIF-1α and HIF-2α in RCC4 ^VHL–/–^ cells) and that *VHL* complementation blocks HIF overexpression as previously described (Supplementary Figure 1). We recently discovered that hypoxia leads to transcriptional down-regulation of FANCD2, a key factor in DNA interstrand crosslink repair and HR [28]. We therefore first performed western blotting for FANCD2 in *VHL*-deficient and *VHL^WT^*-complemented 786-O renal cell carcinoma cells after exposure to normoxia or hypoxia (<0.01% O_2_) for 24 and 48 h (Figure 1A). Consistent with our prior observations, we found that hypoxia led to a reduction in FANCD2 protein levels, though with significant differences between the *VHL–/–* and *VHL^WT^* cells. Under normoxic conditions, we observed 20–30% lower FANCD2 levels in 786-O^VHL–/–^ cells compared to 786-O+VHL^WT^ cells. However, upon hypoxic exposure, FANCD2 underwent greater repression in the 786-O+VHL^WT^ cells compared to the 786-O^VHL–/–^ cells such that FANCD2 levels equalized after 48 h of hypoxia (Figure 1A). We repeated this experiment in the RCC4 matched-pair cell lines and similarly found a 30–40% reduction in FANCD2 levels in RCC4^VHL–/–^ cells compared to RCC4+VHL^WT^ cells under normoxic conditions, with FANCD2 levels equalizing between the two cell lines after 48 h of hypoxia (Figure 1B).

The baseline reduction in FANCD2 that we observed in the *VHL–/–* 786-O and RCC4 cell lines, along with the equalization of FANCD2 levels in the *VHL–/–* and *VHL^WT^* cells under hypoxia, suggests that *VHL* loss may induce changes in DNA repair in a manner similar to hypoxic stress. To test this hypothesis, we examined the expression of additional HR and MMR DNA repair proteins known to be down-regulated by hypoxia. We found that, like FANCD2, the HR proteins BRCA1 and RAD51, as well as the MMR protein MLH1, were all expressed at significantly lower levels in the *VHL*-deficient cells compared to the *VHL^WT^*-complemented cells (Figure 1C). We next examined whether the regulation of these DNA repair genes occurred at the mRNA level. Using quantitative real-time PCR (qRT-PCR) analysis, we found 30–60% reductions in *FANCD2*, *BRCA1*, *RAD51*, and *MLH1* mRNA levels in 786-O^VHL–/–^ and RCC4^VHL–/–^ cells compared to their *VHL^WT^*-complemented matched pairs (Figure 1D).

**Figure 2:**
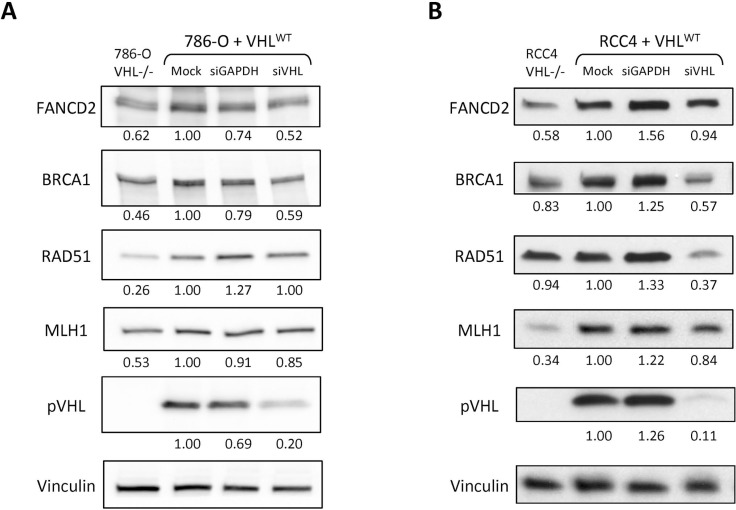
Depletion of pVHL leads to down-regulation of HR and MMR protein expression in renal carcinoma cells Western blotting was performed to analyze FANCD2, BRCA1, RAD51, and MLH1 expression in 786-O+VHL^WT^ cells 48 h post-transfection (**A**) or in RCC4+VHL^WT^ cells 72 h post-transfection (**B**) with GAPDH or VHL siRNA or mock treatment. Non-transfected 786-O^VHL–/–^ and RCC4^VHL–/–^ cells are included for comparison. Vinculin is presented as loading control and numbers below the blots indicate band density relative to vinculin and normalized to VHL^WT^ mock treatment samples.

To attribute the down-regulation of HR and MMR expression in the *VHL*-deficient cells directly to loss of pVHL, we utilized *VHL* siRNA to transiently deplete the 786-O+VHL^WT^ and RCC4+VHL^WT^ cells of pVHL. We then performed western blotting for HR and MMR proteins, and found that pVHL depletion recapitulated the down-regulation of FANCD2, BRCA1, RAD51, and MLH1 seen with *VHL* deficiency in one or both cell lines (Figure 2A, 2B). Previous studies have found that loss of pVHL can lead to altered cell cycle progression [43, 48, 49]. Importantly, HR can only occur in S and G2/M phases when a sister chromatid is present, and several HR genes are known to be differentially expressed throughout the cell cycle [50–53]. We therefore performed cell cycle analysis with propidium iodide staining and a BrdU pulse in the 786-O and RCC4 matched pair cell lines to determine whether differences in cell cycle phase could underlie the changes in HR gene expression. In the 786-O pair, we found a 10% increase in the G1 phase and 7% decrease in the S phase populations in the *VHL*-deficient compared to *VHL^WT^*-complemented cells (Supplementary Figure 2A). In the RCC4 pair, we observed similar but smaller changes, with a 6% increase in the G1 phase and 4% decrease in the S phase populations in the *VHL*-deficient compared to *VHL^WT^*-complemented cells (Supplementary Figure 2B). These changes in cell cycle could partially account for reduced HR gene expression in *VHL*-deficient cells, but are unlikely to play a predominant role given their small size, especially in the RCC4 pair.

### *VHL* loss impairs cellular DNA double-strand break repair by homologous recombination and induces sensitivity to DNA damaging agents

**Figure 3:**
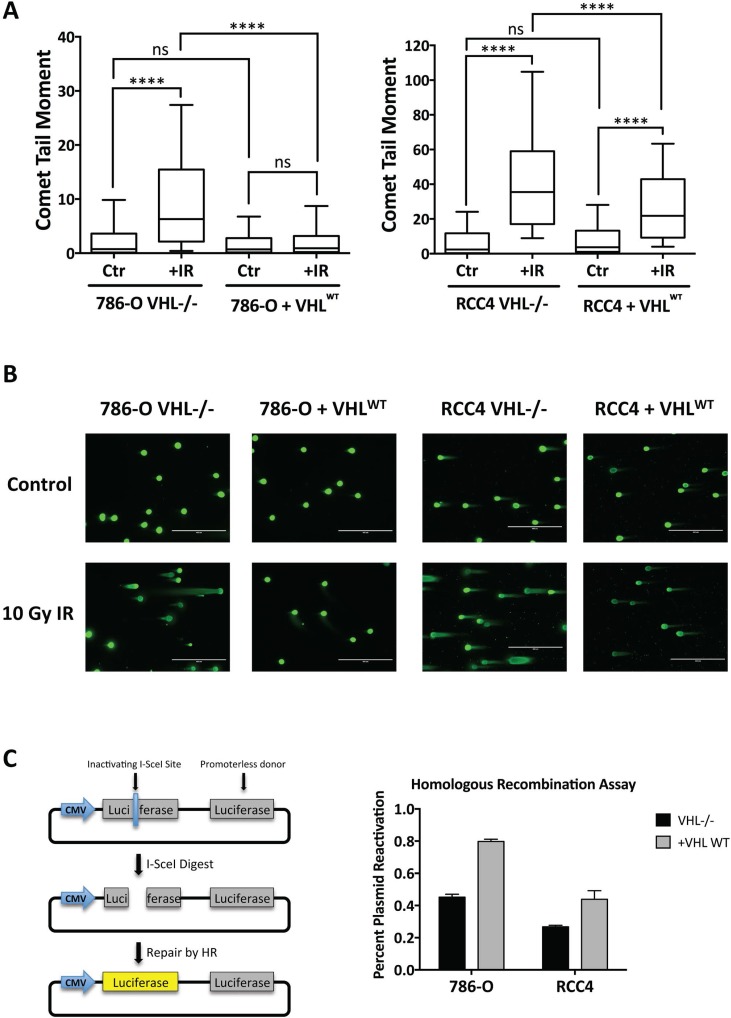
DNA double-strand break repair is impaired in *VHL*-deficient renal carcinoma cells (**A**) The neutral comet assay was performed in 786-O and RCC4 matched pair cells 24 h post-treatment with 10 Gy IR (+IR) or mock treatment (Ctr). Median comet tail moments from analysis of ≥100 comets/sample are presented as box-and-whisker plots. *Boxes*, lower and upper quartiles; *middle line*, median; *whiskers*, 10^th^ to 90^th^ percentiles; ^****^ significant at *p* < 0.0001; ns, not significant. (**B**) Representative images of comets observed in 786-O and RCC4 matched pair cells 24 h post-treatment with 10 Gy IR. (**C**) HR activity was measured in 786-O and RCC4 matched pair cells using the HR luciferase plasmid reactivation assay. *Columns*, mean of 3 replicates; *bars*, SEM.

We next wanted to investigate the functional significance of the relative decrease in expression of HR factors in *VHL*-deficient RCC cells. We started by measuring the repair of DNA double-strand breaks induced by ionizing radiation (IR) using the neutral comet assay. The 786-O and RCC4 matched pair cell lines were treated with 10 Gy IR and assayed 24 h post-irradiation. In both cell lines, we observed a significantly higher median comet tail moment in the irradiated *VHL*-deficient cells compared to the irradiated *VHL^WT^*-complemented cells, indicative of more DNA double-strand breaks persisting 24 h post-irradiation and less efficient repair in the *VHL*-deficient cells (Figure 3A, 3B). In contrast, there were no significant differences between the mean tail moments at baseline in either matched pair of cells. Using additional metrics of analysis of the comet assay data, we found that the mean comet tail moment as well as the median and mean percentages of DNA in the comet tail were significantly higher in the 786-O^VHL–/–^ and RCC4^VHL–/–^ cells compared to the 786-O+VHL^WT^ and RCC4+VHL^WT^ cells 24 h post-irradiation (Supplementary Figure 3), consistent with our conclusion that *VHL*-deficient cells have compromised repair of DNA double-strand breaks.

To assess directly the homologous recombination pathway of DNA double-strand break repair, we utilized a plasmid-based host cell reactivation assay that has been previously characterized [54]. The plasmid used in this assay contains a promoter-driven firefly luciferase gene with an inactivating I-SceI restriction enzyme site and a second promoter-less wild type copy of the firefly luciferase gene. The plasmid is digested with I-SceI enzyme to induce a double-strand break in the luciferase gene and then transfected into cells to allow repair of the break within the cell using the homologous wild type luciferase gene as a template. HR repair efficiency is measured 48 h post-transfection by the percent reactivation of luciferase activity. We conducted this experiment in the 786-O and RCC4 matched pair lines and found a decrease of approximately 50% in plasmid reactivation in the *VHL*-deficient cells relative to the *VHL^WT^*-complemented cells, indicating a significant impairment in HR in the *VHL*-deficient cells (Figure 3C).

**Figure 4:**
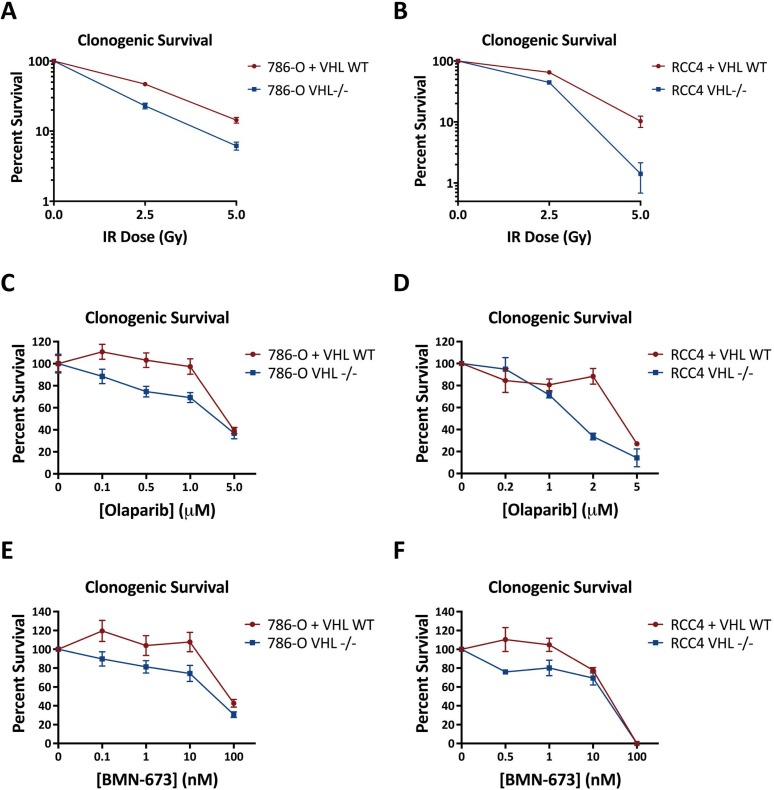
*VHL*-deficient renal carcinoma cells are hypersensitive to IR and PARP inhibitors compared to *VHL*^WT^-complemented cells (**A**, **B**) Clonogenic survival in 786-O^VHL–/–^ and 786-O+VHL^WT^ cells (A) or RCC4^VHL–/–^ and RCC4+VHL^WT^ cells (B) treated with ionizing radiation. (**C**, **D**) Clonogenic survival in 786-O^VHL–/–^ and 786-O+VHL^WT^ cells (C) or RCC4^VHL–/–^ and RCC4+VHL^WT^ cells (D) exposed continuously to the PARP inhibitor Olaparib. (**E**, **F**) Clonogenic survival in 786-O^VHL–/–^ and 786-O+VHL^WT^ cells (E) or RCC4^VHL–/–^ and RCC4+VHL^WT^ cells (F) exposed continuously to the PARP inhibitor BMN-673. *Points*, mean of 3 to 6 replicates; *error* *bars*, SEM.

Based on our finding of impaired homologous recombination in the *VHL*-deficient renal lines, we hypothesized that *VHL* loss would sensitize renal cells to DNA damaging agents and PARP inhibitors. We first performed clonogenic survival assays in the 786-O and RCC4 matched pair lines following irradiation and found that the 786-O^VHL–/–^ and RCC4^VHL–/–^ cells demonstrated increased sensitivity to IR compared to their *VHL^WT^*-complement matched pairs (Figure 4A, 4B). Next, we performed clonogenic assays using the clinically-approved PARP inhibitor, Olaparib, and again found increased sensitivity in the 786-O^VHL–/–^ and RCC4^VHL–/–^ cells compared to the 786-O+VHL^WT^ and RCC4+VHL^WT^ cells (Figure 4C, 4D). We repeated these experiments with a second PARP inhibitor, BMN-673, and found similar increased sensitivity in the 786-O^VHL–/–^ and RCC4^VHL–/–^ cells compared to the 786-O+VHL^WT^ and RCC4+VHL^WT^ cells (Figure 4E, 4F).

### *VHL* deficiency in human clear cell renal carcinoma samples is associated with reduced HR and MMR gene expression

**Figure 5:**
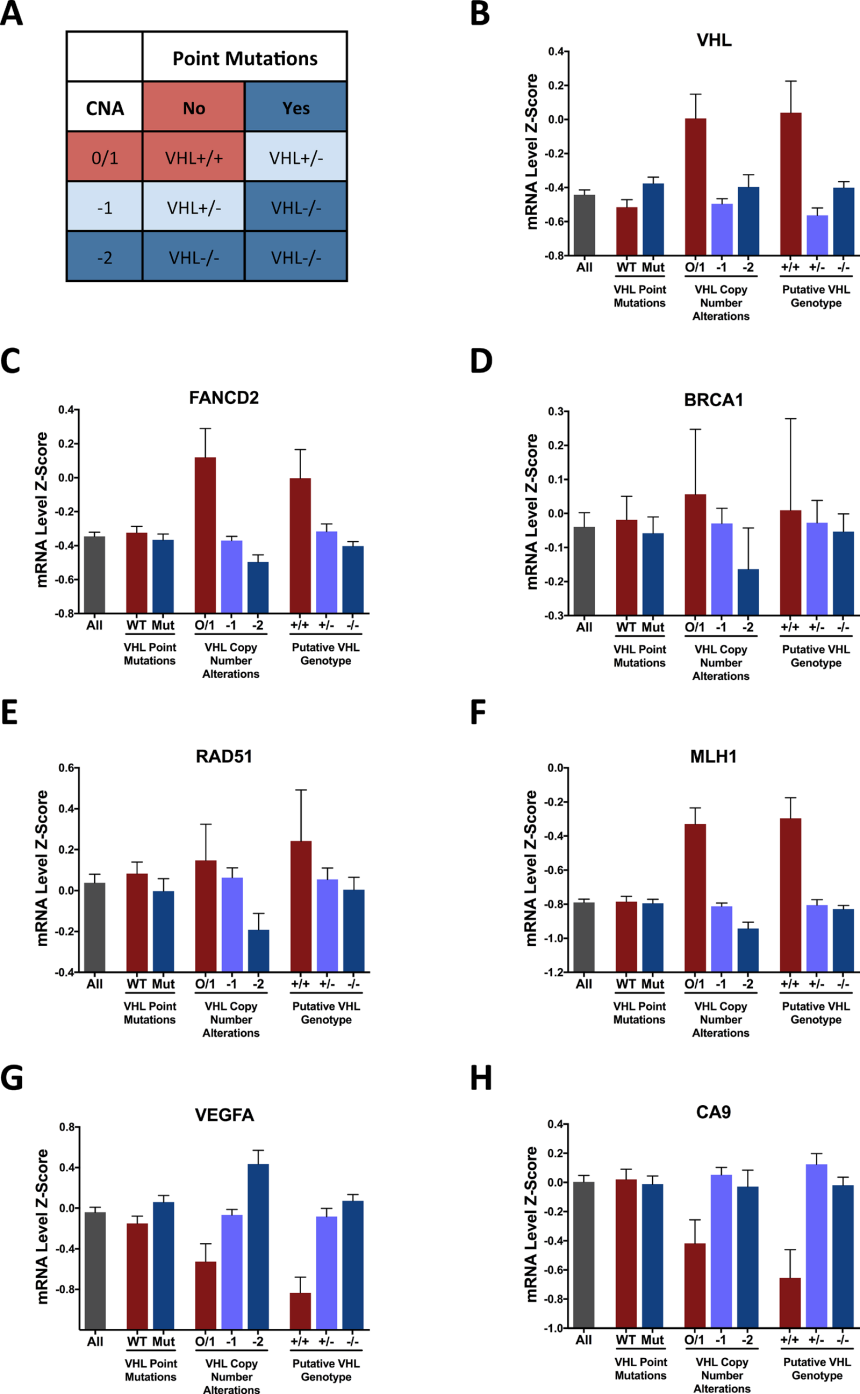
Expression of HR and MMR genes correlates with *VHL* status in human renal clear cell carcinoma samples (**A**) Classification of *VHL* genotype based on *VHL* point mutations and copy number alterations (CNA). (**B**–**H**) mRNA expression levels of indicated genes determined by RNA-sequencing of 418 tumor samples in TCGA Provisional Kidney Renal Clear Cell Carcinoma dataset, grouped on the basis of *VHL* point mutations, *VHL* copy number alterations, or putative *VHL* genotype. *Columns*, mean; *bars*, SEM.

To extend our findings of impaired homologous recombination in *VHL*-deficient cancer cell lines to human tumors, we interrogated The Cancer Genome Atlas (TCGA) Kidney Renal Clear Cell Carcinoma database to analyze DNA repair gene expression in *VHL*-deficient and *VHL^WT^* human renal tumor samples. *VHL* deficiency in renal carcinomas can occur via inactivating mutations or allelic loss. We therefore sorted the tumor samples into groups with and without *VHL* point mutations and groups with copy number alterations of 0/1, -1, or -2. In addition, we determined the putative *VHL* genotype of each tumor sample based on the combination of point mutations and copy number alterations and sorted the samples into homozygous WT (+/+), heterozygous (+/–), and homozygous mutant (–/–) groups (Figure 5A). We first analyzed *VHL* mRNA levels and found reduced expression in the samples with copy number loss, but not with point mutations, as expected (Figure 5B). We then analyzed the mRNA expression of the HR and MMR genes that we found to be reduced in the *VHL*-deficient cell lines, *FANCD2*, *BRCA1*, *RAD51*, and *MLH1*, and found an association between *VHL* inactivation and reduced mRNA expression of these four genes in each grouping method (Figure 5C–5F). Finally, as controls, we analyzed the mRNA expression of two hypoxia-inducible genes, *VEGFA* and *CA9,* and observed increased expression in the *VHL*-deficient tumor samples as expected (Figure 5G–5H).

## DISCUSSION

In this study we have discovered a novel DNA repair deficiency in *VHL*-deficient clear cell renal carcinoma. *VHL*-deficient ccRCC cells have reduced expression of the HR genes, *BRCA1*, *RAD51*, and *FANCD2*, as well as the MMR gene *MLH1* at the protein and mRNA levels compared to *VHL^WT^*-complemented cells. These changes in expression are phenocopied by pVHL depletion and are recapitulated in mRNA sequencing data from a large cohort of human ccRCC tumor samples. Importantly, the decrease in HR gene expression is associated with reduced repair of IR-induced DNA double-strand breaks and by diminished HR activity in a luciferase plasmid reactivation assay in *VHL*-deficient cells. The decrease in HR capacity in *VHL*-deficient cells is further accompanied by increased sensitivity to PARP inhibition compared to *VHL^WT^*-complemented cells. Our results expand the current knowledge of DNA repair regulation in cancer and identify a potential therapeutic target in *VHL*-deficient renal carcinoma.

The down-regulation of HR and MMR expression that we observe in *VHL*-deficient ccRCC cells is similar to the effect of hypoxic stress on DNA repair. Hypoxia regulates DNA repair through many different mechanisms, and it is likely that many of the same processes occur upon loss of *VHL* due to the constitutive activation of hypoxia-induced signaling pathways. Hypoxia induces changes in DNA repair at the transcriptional level via several intermediate transcription factors, including p130/E2F, Myc, DEC1/2, and Sp1, most of which have been shown to be active in *VHL*-deficient renal cells [55–57]. Hypoxia also affects microRNA expression patterns, which can in turn regulate DNA repair protein expression, and at least one of the key hypoxia-induced microRNAs, miR-210, is also overexpressed in *VHL*-deficient renal cells [58]. At the epigenetic level, hypoxia induces changes in histone modifications and DNA methylation that have been shown to repress DNA repair genes, and many studies have described significant changes in the epigenome of *VHL*-deficient renal cells as well [59–62]. Of note, many of the changes in gene expression induced by hypoxia have been shown to be independent of HIF-1 [25, 26, 28–30]. In this study, we utilized two different *VHL*-deficient ccRCC cell lines, one of which overexpresses HIF-1 and one of which has lost HIF-1 expression, obtaining comparable results in both cell lines. Therefore, we conclude that HIF-1 is also dispensable for changes in DNA repair gene expression in *VHL*-deficient RCC, and that HIF-2 may play the more important role.

As with hypoxia, we find that *VHL*-mediated down-regulation of HR genes leads to a functional defect in DNA double-strand break repair by HR. Although a role for *VHL* in DNA repair has not been studied as intensely as its role in angiogenesis and promotion of tumor growth, there have been several studies that support a connection between *VHL* and DNA repair. First, the *VHL* isoform pVHL_19_ has been found to localize to the nucleus and to be up-regulated in response to DNA damage [63, 64]. pVHL has also been shown to localize to the mitotic spindle and prevent misorientation of the spindle during mitosis [45]. Metcalf *et al.* also described a direct role for *VHL* in HR that is dependent upon SOCS1-mediated ubiquitination of pVHL in response to double-strand breaks and pVHL relocalization to nuclear foci [46]. In that study, however, the mechanism by which pVHL promoted HR was not determined. Our finding of decreased HR gene expression in *VHL*-deficient cells could explain the DNA double-strand break repair defect, though additional mechanisms of HR regulation by pVHL cannot be excluded. Interestingly, Schults *et al.* reported a decrease in nucleotide excision repair (NER) capacity using a luciferase reporter reactivation assay in *VHL*-deficient renal cells [47]. Hypoxia has been shown to have a similar effect on NER [65], further supporting the connection between hypoxia-induced and *VHL*-deficiency-induced DNA repair repression.

The term “BRCAness” has been coined to describe tumors with a defect in DNA double-strand break repair by homologous recombination in the absence of *BRCA1* or *BRCA2* mutations [66, 67]. BRCAness can arise from defects in other individual genes involved in HR or its regulation, epigenetic silencing of HR genes, or other non-genetic changes that lead to reduced HR such as hypoxia. Importantly, tumors with BRCAness display hallmark sensitivity to chemotherapy agents that induce replicating fork stalling such as PARP inhibitors and DNA crosslinking agents [68]. Our findings of decreased HR gene expression, impaired DNA double-strand break repair capacity, and sensitivity to PARP inhibitors indicate that *VHL*-deficient renal carcinoma shares some features with BRCAness tumors. However, renal cancer is known to be highly resistant to radiotherapy and cytotoxic chemotherapy, and advanced ccRCC, found in about a third of patients at diagnosis, has an extremely poor 5-year survival rate of about only 10% [69]. Hence, pro-survival pathways likely dominate the aggressive natural history of ccRCC, suggesting that new targeted therapies designed to better exploit potential vulnerabilities in ccRCC are needed.

Current first- and second-line treatment of ccRCC consists of targeted therapy with VEGF receptor tyrosine kinase inhibitors and immunotherapy with IFN-α [70]. Despite these new therapies, however, the majority of patients have progressive disease or relapse, with a median progression-free survival of about only 10 months [69]. Interestingly, prior to the development of these targeted agents, replication stalling agents (5-fluorouracil) and DNA cross-linking agents (mitomycin C, cisplatin) were found to be more effective than other types of chemotherapy [71, 72]. Potentially, the HR defect that we have identified in *VHL*-deficient cells could contribute to this finding and provide a rationale for treatment with drugs displaying hallmark sensitivity in BRCAness tumors. Given the crucial need for better treatment options in RCC, we believe that our findings support the investigation of PARP inhibitors in *VHL*-deficient human renal cell carcinoma.

In conclusion, our findings elucidate a novel connection between hypoxia-induced repression of DNA repair and *VHL* loss in renal cell carcinoma. These findings have important implications for renal carcinoma, but may also be applicable to other cancer types. Synergism between hypoxia-induced repression of HR and PARP inhibitors has been demonstrated *in vitro* [41, 42, 73], and is currently being tested in human clinical trials through the combination of an angiogenesis inhibitor to induce hypoxia and a PARP inhibitor [74–76]. This treatment strategy is growing increasingly promising as a clinical trial of combination therapy with the VEGF inhibitor Cediranib and the PARP inhibitor Olaparib showed efficacy in a cohort of breast cancer patients, both with and without BRCA mutations [76]. Our data suggest that activation of hypoxia-induced signaling pathways, even in the absence of low oxygen, can sensitize cells to PARP inhibitors. We anticipate that this finding will lead to new treatment strategies that take advantage of induced DNA repair deficiencies.

## MATERIALS AND METHODS

### Cell culture and hypoxia

786-O cell lines stably expressing pRC-HA-VHL (786-O+VHL^WT^) or pRC vector alone (786-O^VHL–/–^) were generously provided by Dr. W.G. Kaelin (Medical Oncology, Dana-Farber Cancer Institute, Boston MA) and were authenticated by short tandem repeat profiling at the Yale DNA Analysis Facility and comparison to the published profile. RCC4 cell lines stably expressing pcDNA3-VHL (RCC4+VHL^WT^) or pcDNA3 vector alone (RCC4^VHL−/−^) were obtained through Sigma-Aldrich from the European Collection of Authenticated Cell Cultures. Cells were grown in high-glucose DMEM supplemented with 10% FBS (Invitrogen). Selection of cells containing the pVHL or empty vector constructs was maintained with 500 μg/mL G418 (American Bio). Hypoxic conditions were established as previously described [28].

### Chemicals

Olaparib (Sigma) in DMSO at 50 mM and BMN-673 (Selleck Chemicals) in DMSO at 10 mM were diluted in DMSO to make 1000X solutions and added directly to cell culture media, with 0.1% DMSO used as a control. 5-Bromo-2′-deoxyuridine (BrdU) (Sigma) at 1 mM in DMSO was diluted directly in cell culture media for a final concentration of 10 μM.

### Western blot analysis

Frozen cell pellets were lysed in AZ lysis buffer (50 mM Tris, 250 mM NaCl, 1% Igepal, 0.1% SDS, 5 mM EDTA, 10 mM Na_4_P_2_O_7_, 10 mM NaF) supplemented with Protease Inhibitor Cocktail (Roche) on ice for 20 min. Cellular debris was cleared by centrifugation and lysate protein concentration was quantified using the DC Protein Assay (Bio-Rad). Equal amounts of protein were subjected to SDS-PAGE in Mini-PROTEAN TGX gradient gels (Bio-Rad) and then transferred to nitrocellulose membrane. The following primary antibodies were used for western blot analysis: mouse monoclonal anti-FANCD2 (103, Abcam), mouse monoclonal anti-BRCA1 (D9, Santa Cruz Biotechnology), mouse monoclonal anti-RAD51 (14B4, Novus Biologicals), mouse monoclonal anti-MLH1 (BD554073, BD Biosciences), mouse monoclonal anti-Vinculin (SPM227, Abcam), mouse monoclonal anti-VHL (BD556347, BD Biosciences), rabbit polyclonal anti-VHL (#68547, Cell Signaling Technology), mouse monoclonal anti-HIF-1α (BD610958, DB Biosciences), and rabbit monoclonal anti-HIF-2α (D9E3, Cell Signaling Technology). Band intensities were quantified using ImageJ software and normalized to Vinculin expression.

### Reverse transcription-quantitative PCR (RT-qPCR)

Total RNA was prepared using the RNeasy Mini Kit (Qiagen). The optional on-column DNase digestion was performed with the RNase-Free DNase Set (Qiagen) to eliminate genomic DNA. Complementary DNA (cDNA) was synthesized using 750 ng RNA in the High Capacity cDNA Reverse Transcription Kit (Applied Biosystems). The resulting cDNA was diluted 1:5 and used in triplicate PCRs containing TaqMan Gene Expression Assay premixed primers and probes for *FANCD2, BRCA1*, *RAD51, MLH1*, and 18S rRNA and TaqMan Fast Universal PCR Master Mix (Applied Biosystems). A StepOnePlus Real-Time PCR System (Thermo Fisher Scientific) was used to measure fluorescence intensity in real-time and to calculate cycle thresholds. C_t_ values were normalized to 18S rRNA and relative expression was calculated using the –ΔΔC_t_ method.

### VHL siRNA depletion

Cells were plated at 10^5^ cells/well, allowed to adhere overnight and then transfected with 50 nM SMARTpool ON-TARGETplus VHL siRNA (Dharmacon) or ON-TARGETplus GAPD Control Pool (Dharmacon) using DharmaFECT 1 Transfection Reagent (Dharmacon). Culture media was replaced 8 h post-transfection and cells were assayed by western blotting 48 h or 72 h post-transfection.

### Comet assay

Cells were plated, allowed to adhere overnight, and treated with 10 Gy IR. Cells were harvested 24 h post-irradiation and resuspended in LM Agarose (Trevigen). Neutral single-cell gel electrophoresis was conducted using the CometAssay Electrophoresis System (Trevigen) according to the manufacturer's protocol. Data were collected with an EVOS FL microscope (Advance Microscopy Group) and analyzed with CometScore software (TriTek Corporation). Statistical analyses were performed with the Kruskal-Wallis one-way ANOVA followed by Dunn's Multiple Comparison Test using GraphPad Prism Version 7.0a for MAC OS X (GraphPad Software).

### Clonogenic survival assays

For IR clonogenic assays, cells were treated with varying doses of IR and immediately reseeded in triplicate at 100, 300, and 1000 cells/well in 6-well plates. For Olaparib and BMN-673 clonogenic assays, cells were seeded in triplicate at 100–500 cells/well in 6-well plates and allowed to adhere overnight. Cells were then treated continuously with varying concentrations of Olaparib or BMN-673. In all assays, cells were cultured for 7–10 days until colonies formed, replacing culture media every 3 days. Cells were permeabilized with 0.9% saline solution and stained with crystal violet in 80% methanol. Colonies with >50 cells were counted manually.

### Homologous recombination plasmid reactivation assay

The assay was performed as previously described using an HR luciferase reporter plasmid: a modified gWIZ.Luciferase vector (Gelantis) with an inactivating I-SceI recognition site 56 amino acids into the firefly luciferase gene and a promoter-less copy of the firefly luciferase open reading frame 700 base pairs downstream as donor template for HR [54]. Briefly, this HR luciferase reporter plasmid was digested with I-SceI restriction enzyme (New England Biolabs) to induce a double-strand break in the firefly luciferase gene. Cells were then transfected with 2 μg of linearized HR luciferase reporter plasmid or 2 μg of unmodified gWIZ.Luciferase vector using Lipofectamine 3000 Reagent (Thermo Fisher Scientific) per manufacturer's protocol. Cells were also co-transfected with 50 ng pCMV-RL (Promega) as a transfection efficiency control. Culture media was replaced 6 h post-transfection, and luciferase activity was measured 48 h post-transfection using the Dual-Luciferase Reporter Assay System (Promega) per manufacturer's protocol. Percent reactivation was calculated by dividing Firefly luciferase activity by Renilla luciferase activity and then normalizing HR luciferase reporter plasmid reactivation to unmodified gWIZ.Luciferase.

### Analysis of the cancer genome atlas (TCGA) database

*VHL* mutation and copy number alteration data, and *VHL, FANCD2, BRCA1, RAD51*, *MLH1, VEGFA* and *CA9* mRNA expression Z-scores (RNA Seq V2 RSEM) for 446 complete tumor samples in the TCGA Provisional Kidney Renal Clear Cell Carcinoma dataset generated by the TCGA Research Network (http://cancergenome.nih.gov) were downloaded via cBioPortal [77, 78]. On the basis of prior pathology re-review or molecular analysis, 28 tumor samples previously identified as likely non-clear cell RCC were excluded from further analysis [79]. The remaining 418 samples were grouped by *VHL* mutation status, *VHL* copy number, or putative *VHL* genotype and mRNA expression of each gene was compared between groups.

## SUPPLEMENTARY MATERIALS FIGURES



## Abbreviations

VHL: von Hippel-Lindau
HR: homologous recombination
NHEJ: non-homologous end joining
ccRCC: clear cell renal cell carcinoma
pVHL: von Hippel-Lindau protein
HIF: hypoxia-inducible factor
PHD: prolyl hydroxylase
MMR: mismatch repair
PARP: poly-ADP-ribose polymerase
NER: nucleotide excision repair
IR: ionizing radiation
TCGA: The Cancer Genome Atlas.
