# Supplementary material for: Suppression of homology-dependent DNA double-strand break repair induces PARP inhibitor sensitivity in VHL-deficient human renal cell carcinoma
Source: Oncotarget. 2017 Dec 19;9(4):4647–60. doi: 10.18632/oncotarget.23470 (PMC5797003; doi:10.18632/oncotarget.23470)
Supplement: Supplementary file 1 [file oncotarget-09-4647-s001.pdf]

# Suppression of homology-dependent DNA double-strand break repair induces PARP inhibitor sensitivity in *VHL*-deficient human renal cell carcinoma

## SUPPLEMENTARY MATERIALS

### Cell cycle analysis

Cells in log-phase growth at approximately 60% confluency were pulsed with 10 µM BrdU for 20 minutes. Cells were immediately trypsinized, washed in 1X PBS, and fixed with 70% ethanol at –20°C overnight. Cells were then incubated in 2 M HCl + 0.5% Triton X-100 for 30 minutes to denature DNA, neutralized with 0.1 M $Na_2B_4O_7$ pH 8.5, washed with 1% BSA + 0.2% Triton X-100 in 1X PBS, and stained with FITC Mouse Anti-BrdU (BD Biosciences) at 4°C overnight. Finally, cells were washed with 1% BSA + 0.2% Triton X-100 in 1X PBS, incubated in PI/RNase Staining Buffer (BD Biosciences) for 15 minutes, and analyzed by flow cytometry on a FACSCalibur Cytometer (BD Biosciences). Data were analyzed using FlowJo software (Tree Star Inc.).

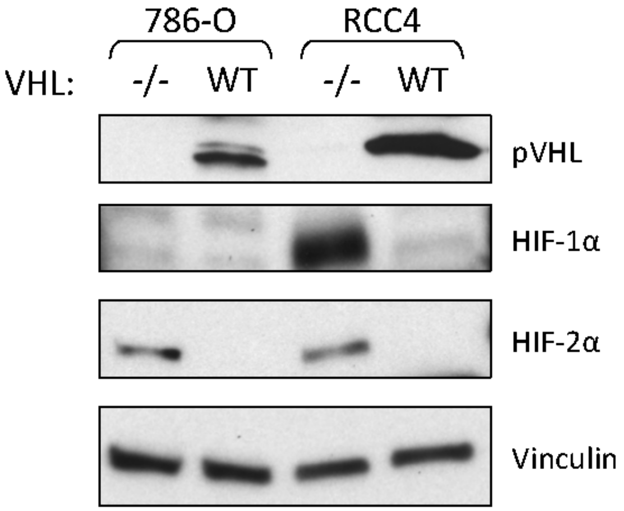


**Supplementary Figure 1: Validation of *VHL–/–* and *VHL$^{WT}$*-complemented 786-O and RCC4 renal cell lines.** Western blotting was performed to analyze pVHL, HIF-1α, and HIF-2α expression in 786-O$^{VHL-/-}$, 786-O+VHL$^{WT}$, RCC4$^{VHL-/-}$, and RCC4+VHL$^{WT}$ cells. Note that 786-O$^{VHL-/-}$ cells overexpress HIF-2α and RCC4$^{VHL-/-}$ cells overexpress both HIF-1α and HIF-2α. Complementation with pVHL in 786-O+VHL$^{WT}$ and RCC4+VHL$^{WT}$ cells counteracts HIF-1α and HIF-2α overexpression.

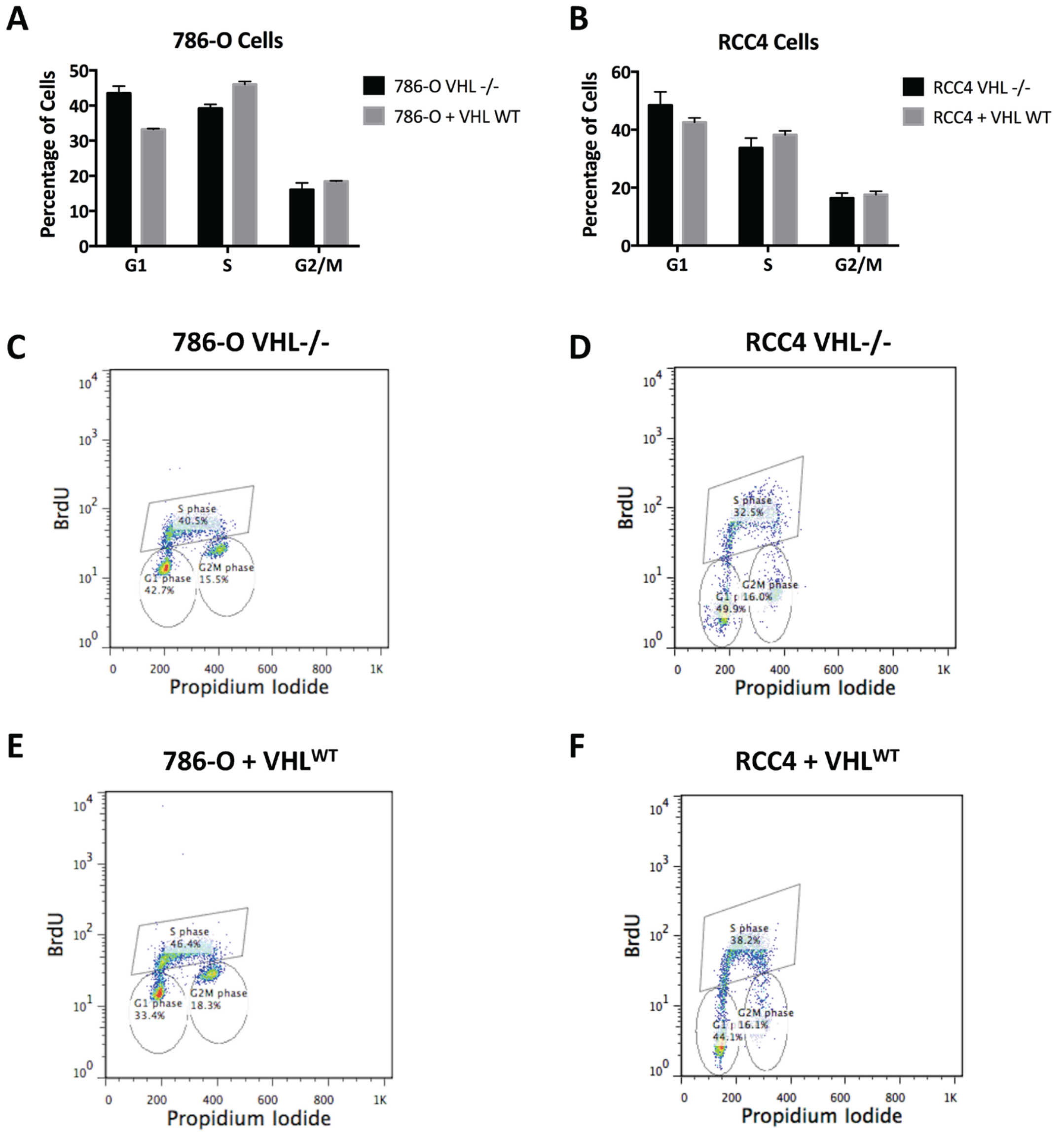


**Supplementary Figure 2: Cell cycle analysis in 786-O and RCC4 matched pair cell lines.** (**A**, **B**) Cell cycle distribution in 786-O$^{VHL-/-}$ and 786-O+VHL$^{WT}$ cells or RCC4$^{VHL-/-}$ and RCC4+VHL$^{WT}$ cells determined by analysis of PI/BrdU FACS plots. *Columns*, mean of 3 to 4 replicates; *bars*, SD. (**C**–**F**) Representative PI/BrdU FACS plots in 786-O$^{VHL-/-}$, 786-O+VHL$^{WT}$, RCC4$^{VHL-/-}$, and RCC4+VHL$^{WT}$ cells.

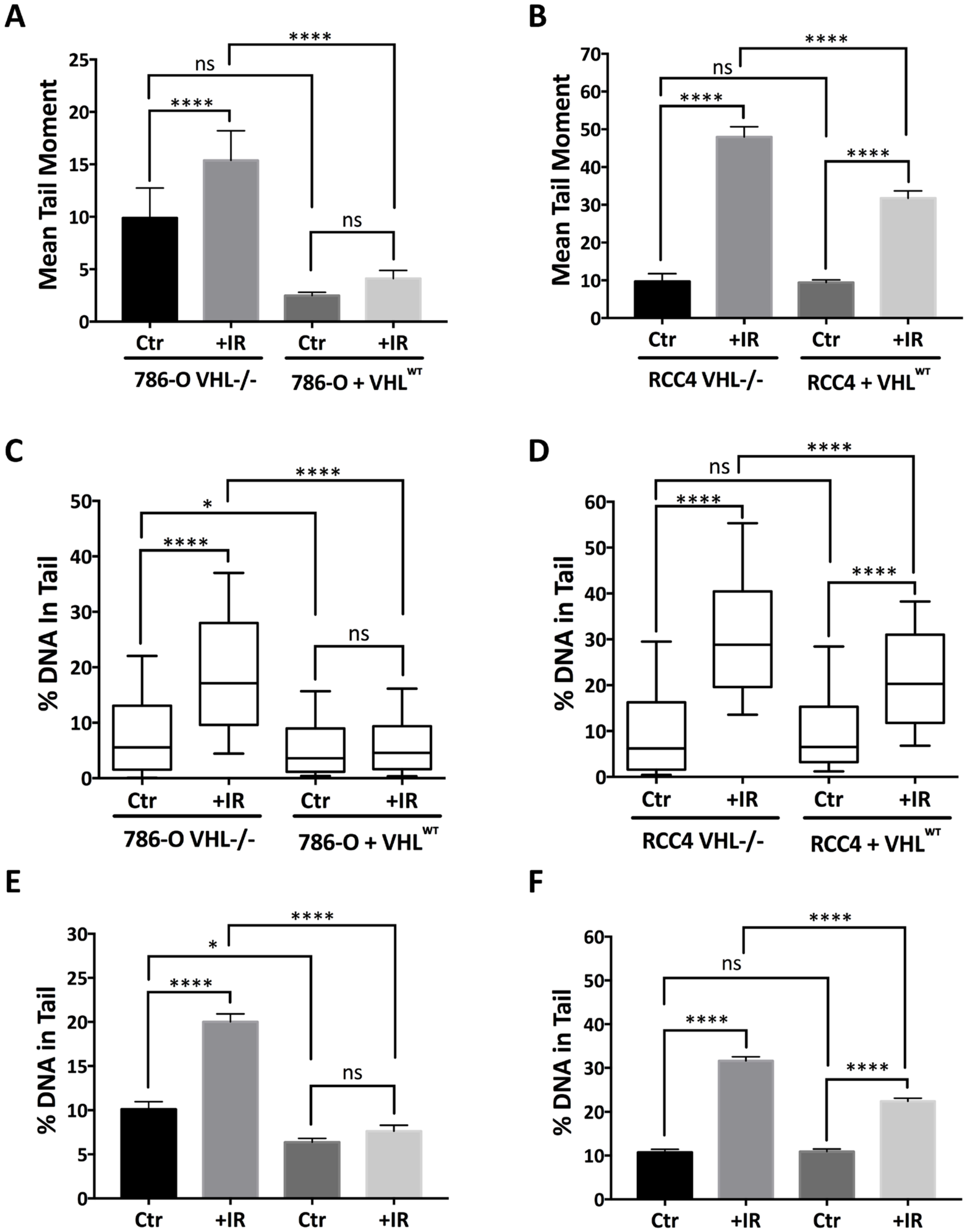


**Supplementary Figure 3: The neutral comet assay performed in 786-O and RCC4 matched pair cells 24 h post-treatment with 10 Gy IR (+IR) or mock treatment (Ctr).** (**A**, **B**) Mean comet tail moments from analysis of ≥100 comets/sample. *Columns*, mean; *bars*, SEM. (**C**, **D**) Median % DNA in the tail from analysis of ≥100 comets/sample presented as box-and-whisker plots. *Boxes*, lower and upper quartiles; *middle line*, median; *whiskers*, 10th to 90th percentiles. (**E**, **F**) Mean % DNA in the tail from analysis of ≥100 comets/sample. *Columns*, mean; *bars*, SEM. For all panels, $^{****}$significant at $p < 0.0001$; $^{*}$significant at $p < 0.05$; ns, not significant.
